# Minimally invasive surgery for intragastric trichobezoar in pediatrics: A case report

**DOI:** 10.1016/j.ijscr.2025.111649

**Published:** 2025-07-10

**Authors:** Mahdi Parvizi Mashhadi, Kristin E. Shipman

**Affiliations:** a Mashhad University of Medical Sciences, Mashhad, Iran; b Cincinnati Children's Hospital, Cincinnati, OH, USA; c Department of Pediatric Surgery, Rocky Mountain Hospital for Children, Denver, Colorado, USA

**Keywords:** Trichobezoar, Pediatric surgery, Laparoscopy, Minimally invasive surgery, Case report, Gastrointestinal obstruction

## Abstract

**Introduction and importance:**

Bezoars are indigestible masses that accumulate in the gastrointestinal tract, with trichobezoars specifically resulting from the ingestion of hair. These masses are most commonly observed in children with psychiatric conditions such as trichotillomania and trichophagia. Symptoms include abdominal pain, vomiting, constipation, and anorexia, with complications ranging from gastric ulcers to obstruction or perforation. Surgical removal is the definitive treatment, and minimally invasive techniques offer advantages over traditional open surgery.

**Case presentation:**

We report the case of a 4-year-old girl with a history of ingesting hair, plastic toys, and cat hair. She presented with vomiting, constipation, anorexia, and abdominal pain but did not experience weight loss or early satiety. Physical examination revealed a palpable upper abdominal mass, and laboratory results were normal. Endoscopic retrieval of the bezoar was attempted but unsuccessful. Given the lack of signs of bowel obstruction, a computed tomography (CT) scan was not performed. The patient underwent laparoscopic evaluation of the small bowel, followed by removal of the bezoar.

**Clinical discussion:**

The laparoscopic removal of the giant trichobezoar in this pediatric patient was successful. The procedure was safe, effective, and resulted in rapid recovery, minimal postoperative discomfort, and excellent cosmetic outcomes. In this case, the minimally invasive approach provided advantages over traditional open surgery, including reduced recovery time and less postoperative pain.

**Conclusion:**

Laparoscopic surgery should be considered a viable alternative to open surgery for the removal of trichobezoars in pediatric patients. The technique is safe, effective, and offers a favorable outcome when performed in appropriately selected cases.

## Highlights

• **Minimally invasive laparoscopic approach** for the removal of a giant gastric trichobezoar in a pediatric patient, showcasing the safety and efficacy of this technique.
• **Successful management** of a 4-year-old girl with a history of trichophagia, presenting with abdominal pain, vomiting, anorexia, and constipation.
• **Four-port laparoscopic technique** utilizing an endo bag to ensure controlled removal and minimize peritoneal contamination, avoiding the risks associated with open surgery.
• **Postoperative outcomes** demonstrating minimal pain, rapid recovery, and an excellent cosmetic result, highlighting the advantages of minimally invasive surgery.
• **Clinical implication** of laparoscopic intervention as a preferred method for the surgical treatment of large trichobezoars in pediatric patients, compared to traditional open surgery.

## Graphical abstract

**Figure:**
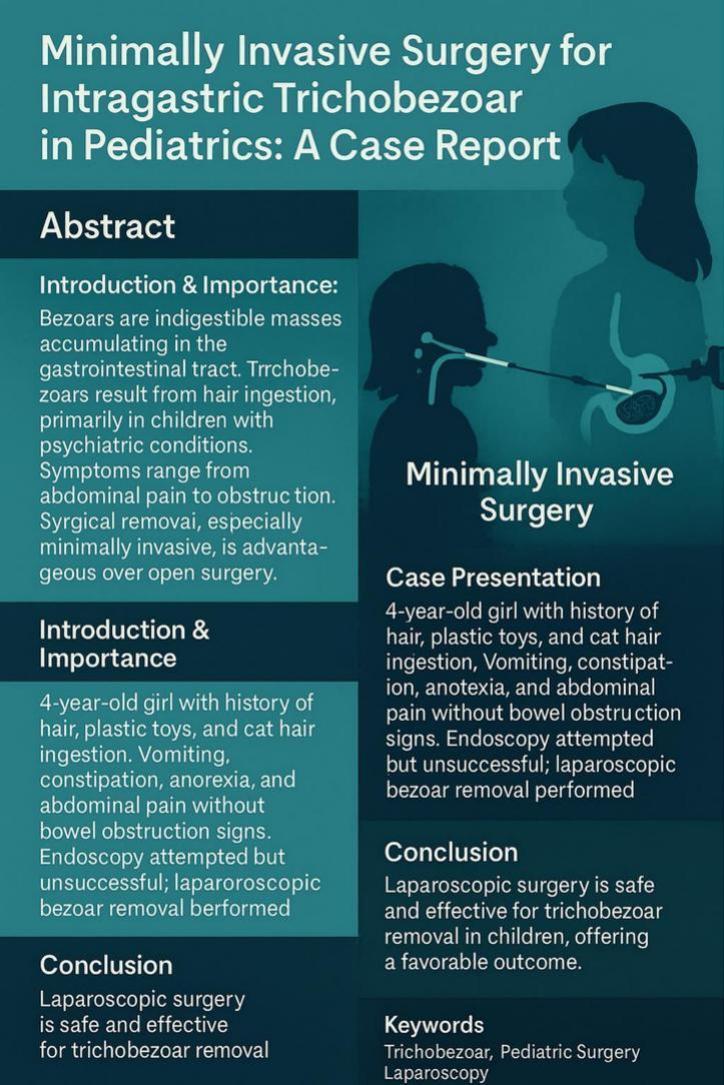

## 1 Introduction

Bezoars are compacted masses of indigestible foreign substances that collect and harden in the gastrointestinal tract, typically within the stomach. Bezoars are categorized into four types based on their composition: trichobezoars, phytobezoars, lactobezoars, and pharmacobezoars [1]. A trichobezoar, also known as a “hair bezoar,” is a rare occurrence often associated with psychiatric conditions such as trichotillomania and trichophagia, which involve the compulsion to pull out one's hair and the desire to consume it, respectively [2]. Typically, a trichobezoar is situated in the stomach, but it can also extend beyond the pylorus into the duodenum and small intestine, a phenomenon known as Rapunzel's syndrome [3].

More than 90 % of cases are in children and young females [4]. The exact occurrence rate of this condition is not well-defined, but according to case studies, 5–30 % of patients with trichotillomania develop trichophagia, and 1–35 % of these cases result in the formation of a trichobezoar [5]. There is no agreement in the literature regarding any link between prevalence and race [6].

Patients with a trichobezoar might either be asymptomatic or exhibit vague, nonspecific symptoms; 80 % commonly report epigastric discomfort [7]. Other potential symptoms include abdominal distension, nausea, vomiting, early feelings of fullness, a sensation of bloating after meals, foul breath, loss of appetite, difficulty swallowing, and unintended weight loss [8]. Significant weight loss is often attributed to early satiety and repeated episodes of vomiting. Gastric bezoars can also lead to complications such as mucosal ulceration from pressure necrosis, which may result in gastrointestinal bleeding and obstruction of the gastric outlet [2]. Additionally, patches of hair loss may be evident, typically as a result of trichotillomania [9].

Surgical treatment is considered the definitive approach for managing gastric and intestinal bezoars. The optimal procedure involves performing a gastrotomy to extract the bezoar, utilizing either an open laparotomic technique or a minimally invasive laparoscopic method.

This case report has been reported in line with the SCARE checklist [10].

## 2 Case report

**Fig. 1:**
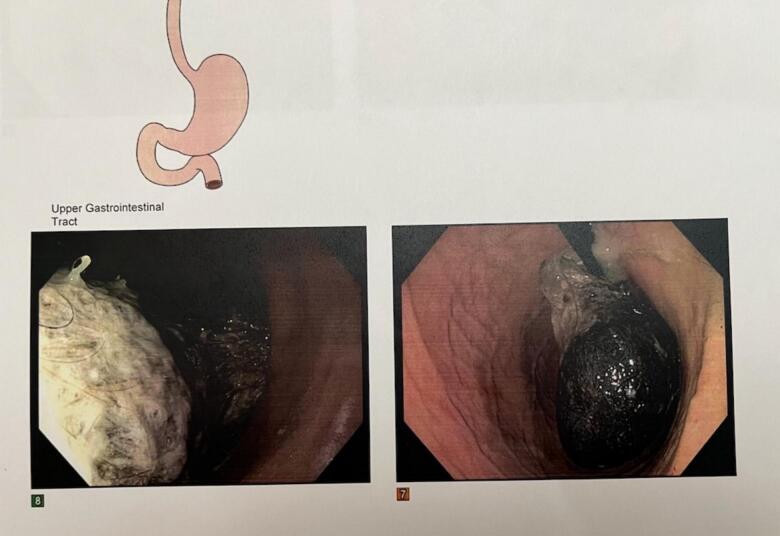
Endoscopic view of a gastric trichobezoar, composed of hair, food, and mucus, occupying the gastric cavity. Endoscopic retrieval was attempted but unsuccessful.

A 4-year-old girl was referred to our clinic for evaluation of a gastric bezoar. Her parents reported that she had ingested her own hair, as well as other hairs, plastic toys, and her cat's hair. She experienced sporadic episodes of vomiting, decreased bowel movements, anorexia, and abdominal pain. She denied weight loss, nausea, and early satiety. The child is under the care of a pediatric gastroenterologist who performed an upper digestive endoscopy. The endoscopic report revealed the presence of a bezoar, which was attempted to be removed but could not be (Fig. 1).

Upon admission, physical examination showed the child to be well-developed and well-nourished for her age. She exhibited no specific abdominal pain or tenderness in the mesogastrium, but a palpable mass was noted in the upper abdomen. Laboratory values were within normal limits. Given that the patient did not present with bowel obstruction symptoms, an abdominal computed tomography (CT) scan was not performed. However, we planned to examine the small bowel laparoscopically. We discussed with the parents that we may potentially remove any foreign body from the small intestine if found upon examination, and they consented.

An upper digestive endoscopy was initially performed, revealing an oval-shaped mass composed of hair, food, and mucus occupying the gastric cavity. The gastric mucosa appeared normal. Due to the size of the mass, endoscopic removal was not possible, and the patient was subsequently prepared for laparoscopic removal.

**Fig. 2:**
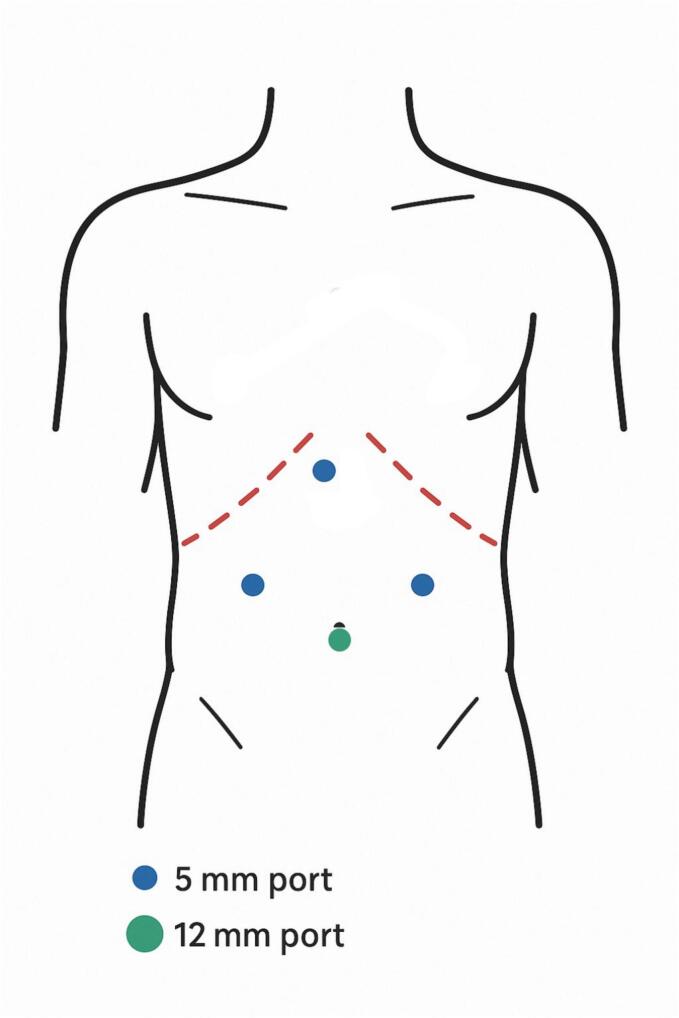
Schematic illustration of laparoscopic port placement facilitating optimal instrument control and safe bezoar extraction.

Under general anesthesia, a 10-mm infraumbilical camera port was inserted, and a pneumoperitoneum of 15 mmHg was established with carbon dioxide. A 30-degree laparoscope was introduced, and a laparoscopic-guided transversus abdominis plane (TAP) block with 20 ml of 0.2 % ropivacaine was administered. Two 5-mm trocars were inserted into the right and left mid-abdomen. During the procedure, an additional 5-mm access was created in the right upper quadrant (RUQ). To insert the endo bag into the stomach, the umbilical port was removed, and a 12-mm laparoscopic retrieval bag was inserted through the same opening. This strategic port configuration enabled precise instrument maneuverability, effective traction and dissection, and safe extraction of the bezoar with minimal gastric wall trauma (Fig. 2).

**Fig. 3:**
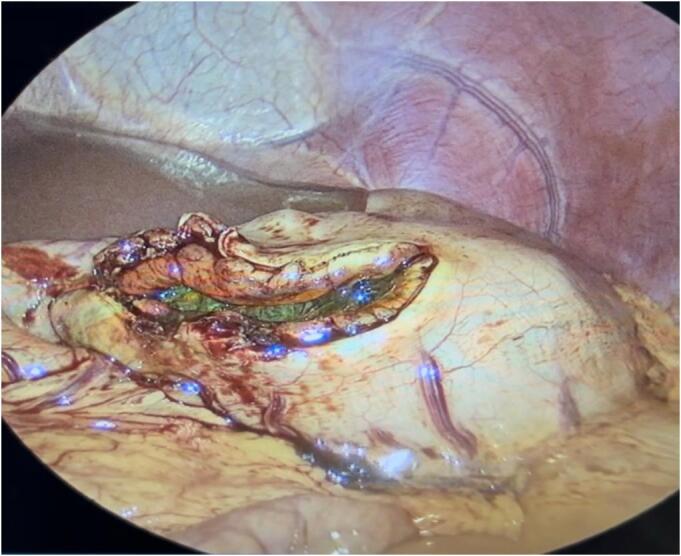
Laparoscopic view of the gastrotomy site, with the trichobezoar inside the stomach. Gentle traction is applied for extraction.

**Fig. 4:**
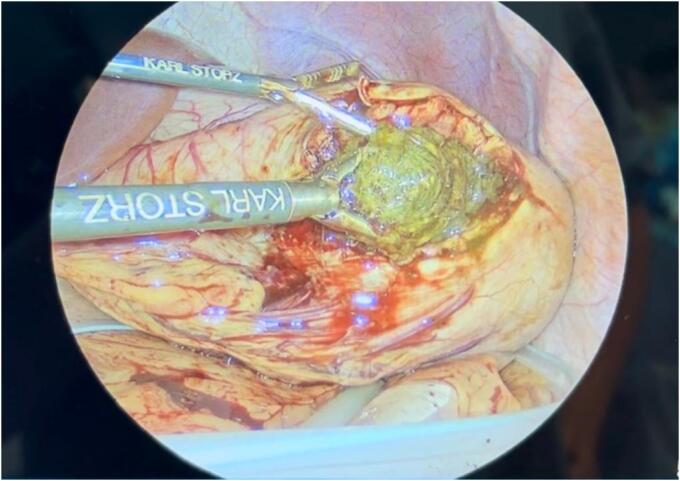
Intraoperative laparoscopic image showing the trichobezoar during its removal from the gastric cavity.

**Fig. 5:**
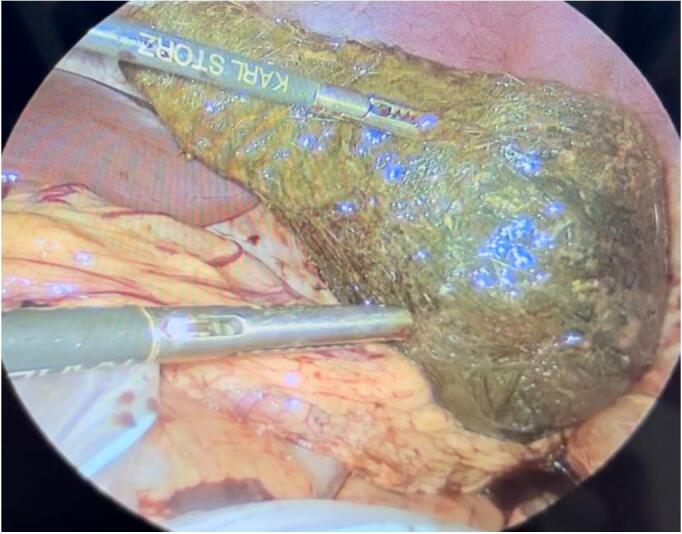
The trichobezoar placed into the laparoscopic retrieval bag for safe and controlled extraction from the stomach.

The surgical procedure was conducted in a structured, stepwise fashion tailored for optimal safety and efficacy in a pediatric patient. Following initial diagnostic laparoscopy, a linear anterior gastrotomy was performed under direct visualization using a 5-mm Maryland Sealer (CoolSeal™). The gastrotomy edges were exteriorized with transabdominal stay sutures to maintain exposure and minimize intraperitoneal contamination (Fig. 3, Fig. 4). The bezoar, occupying the majority of the gastric lumen, was extracted intact and immediately enclosed within a 12-mm laparoscopic retrieval bag (USSC Endo Catch II; U.S. Surgical Corp) introduced through the umbilical port. After secure containment, the specimen was gradually morcellated extracorporeally to facilitate removal through the same incision (Fig. 5). Gastric closure was performed using a linear stapler with intraoperative leak testing via nasogastric insufflation to confirm integrity. This multi-port laparoscopic technique enabled effective management with minimal manipulation, reduced contamination risk, and excellent visualization.

**Fig. 6:**
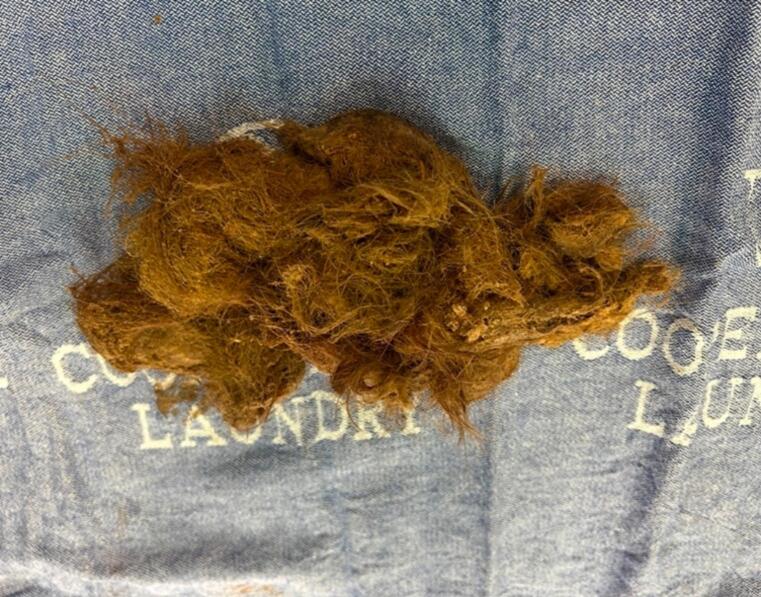
Postoperative image demonstrating the fragmented trichobezoar being carefully removed from the retrieval bag through the umbilical incision.

The neck of the endo bag was then externalized through the umbilical incision, and Kocher's forceps were used to gradually fragment the trichobezoar (Fig. 6). The entire operation took approximately 2.5 h. The nasogastric tube was removed on the first postoperative day. She remained NPO. On POD#3, a leak study showed normal emptying with no leak, and oral feeding was initiated.

## 3 Discussion

Trichobezoars are a rare but important clinical finding, especially in pediatric patients with psychiatric conditions such as trichotillomania and trichophagia. Diagnosis often proves challenging, as symptoms may be nonspecific, and imaging studies such as X-rays and ultrasounds may not provide sufficient evidence [11]. Advanced imaging techniques, including upper gastrointestinal series and CT scans, can assist in the diagnosis, with CT scans showing a heterogeneous intraluminal mass interspersed with gas, creating a filling defect in the stomach [12]. However, upper gastrointestinal endoscopy remains the most reliable diagnostic tool, allowing direct visualization of the bezoar and assessing for complications such as mucosal erosion or ulceration [13].

The treatment of trichobezoars is primarily surgical, particularly for larger bezoars. While endoscopic retrieval is a first-line option for smaller bezoars, larger masses often require surgical intervention. The optimal surgical technique depends on the size, location, and composition of the bezoar. Laparotomy has traditionally been used for large bezoars, but minimally invasive laparoscopic techniques are gaining favor due to their benefits, including reduced postoperative pain, shorter recovery times, and improved cosmetic outcomes [15,16].

Single-port endoscopy or transoral endoscopic retrieval was deemed suboptimal in this case due to the bezoar's considerable size, firm consistency, and complete occupation of the gastric lumen. Endoscopic attempts had previously failed due to the density and adherence of the mass. Furthermore, the risk of mucosal trauma, incomplete retrieval, and intra-gastric fragmentation—raising the potential for distal obstruction or recurrence—necessitated a controlled laparoscopic approach. The multi-port technique provided superior access, bimanual manipulation, and ensured safe, intact extraction without intraperitoneal spillage, which is a known concern in endoscopic or single-port attempts for large bezoars [17].

This report contributes novel technical insights into the management of large trichobezoars in very young pediatric patients using a fully laparoscopic approach. While laparotomy remains the standard in many centers, our case demonstrates that minimally invasive surgery is not only feasible but also advantageous in select patients under five years of age. Specific refinements—such as tailored port placement, gastrotomy edge suspension, and en bloc retrieval using endobags—underscore critical steps that can reduce complications, operative time, and recovery duration [18]. By detailing this operative strategy and its outcome, the present case adds to the limited literature supporting safe laparoscopic management of complex gastric bezoars in early childhood.

Our approach, using a four-port method with a retrieval bag, proved effective and minimized the risk of complications, leading to a successful outcome. The operative time was approximately 2.5 h, with one hour spent on fragmentation and removal of the bezoar. The patient had a smooth recovery, with no postoperative complications, and excellent cosmetic results.

## 4 Conclusion

In conclusion, laparoscopic intragastric removal of a trichobezoar in pediatric patients is a safe and effective procedure. It offers significant advantages over traditional open surgery, including reduced risk of contamination, improved recovery, and excellent cosmetic outcomes. This technique should be considered the treatment of choice for appropriately selected patients.

## Consent

Written informed consent for publication of this case report and any accompanying images was obtained from the patient's parents or legal guardian. Documentation of the consent is securely stored and is available for review by the Editor-in-Chief of this journal upon request, ensuring compliance with ethical standards and patient confidentiality.

## Ethical approval

Informed consent was obtained from the patient's parents for publication of this case report and any accompanying images.

## Funding

This research did not receive specific funding from any agency.

## Author contribution

**Mahdi Parvizi Mashhadi, MD**: Study concept and design, data collection, data analysis and interpretation, writing the paper – original draft, writing the paper – review & editing, supervision.

**Kristin E. Shipman, MD**: Data analysis and interpretation, writing the paper – review & editing, validation, supervision.

## Guarantor

**Mahdi Parvizi Mashhadi, MD** accepts full responsibility for the work and the conduct of the study, had access to the data, and controlled the decision to publish.

## Research registration number

This study was not registered as it is not a 'First in Man' study and registration was not required.

## Conflict of interest statement

The authors declare no conflicts of interest.
